# Population dynamics and habitat preferences of *Phlebotomus orientalis* in extra-domestic habitats of Kafta Humera lowlands – kala azar endemic areas in Northwest Ethiopia

**DOI:** 10.1186/1756-3305-7-359

**Published:** 2014-08-06

**Authors:** Wossenseged Lemma, Habte Tekie, Meshesha Balkew, Teshome Gebre-Michael, Alon Warburg, Asrat Hailu

**Affiliations:** Department of Parasitology, School of Biomedical and Laboratory Sciences, College of Medicine and Health Sciences, University of Gondar, P.O. box: 196, Gondar, Ethiopia; Department of Zoology, Faculty of Life Science, Addis Ababa University, Addis Ababa, Ethiopia; Aklilu Lemma Institute of Pathobiology, Addis Ababa University, Addis Ababa, Ethiopia; Department of Microbiology & Molecular Genetics, The Institute for Medical Research Israel-Canada, The Kuvin Centre for the Study of Infectious & Tropical Diseases, The Hebrew University – Hadassah Medical School, The Hebrew University of Jerusalem, Jerusalem, 91120 Israel; Department of Microbiology, Immunology & Parasitology, Faculty of Medicine, Addis Ababa University, Addis Ababa, Ethiopia

**Keywords:** Population dynamics, Habitat preferences, *P. orientalis*, Extra-domestic habitats, Kafta Humera lowlands, Kala-azar

## Abstract

**Background:**

Kafta Humera lowlands are endemic for kala-azar (visceral leishmaniasis). These lowlands are characterized by black clay soil which is used for growing sesame, sorghum and cotton for commercial purposes.

The aim of this study was to determine seasonal dynamics and habitat preferences of *Phlebotomus orientalis,* the vector of kala-azar*,* in extra-domestic habitats of Kafta Humera lowlands.

**Methods:**

CDC-light Trap [CDC-LT] and Sticky paper Trap [ST] were used to collect sand flies from different habitats before species identification by their morphological characteristics using appropriate keys. Data summarized and analyzed included: species, sex, density, habitats, type of trap used and date (month).

**Results:**

A total of 389,207 sand flies using CDC-LT (n = 955) and ST (n = 5551) were collected from May 17, 2011 to June 6, 2012. The highest Mean Monthly Density (MMD) of *P. orientalis* trapped by CDC-LT was found in thickets of *Acacia seyal* in March (64.11 ± 75.87). The corresponding highest MMD of *P. orientalis* trapped by STs was found in April (58.69 ± 85.20) in agricultural field. No *P. orientalis* were caught in September using CDC traps and July-October using sticky traps. The overall MMD of *P. orientalis* trapped by CDC-LT was 15. 78 ± 28.93 (n = 320) in agricultural field, 19.37 ± 36.42 (n = 255) in thickets of *A. seyal,* and 3.81 ± 6.45 (n = 380) in dense mixed forest. Similar habitats in different localities did not show statistically significant difference for the MMD of *P. orientalis* trapped by CDC-LT (p = 0.117) and ST (p = 0.134).

**Conclusion:**

Agricultural fields and thickets of *A. seyal* habitats, which exhibit extensive soil cracks and fissures, as opposed to dense mixed forests, serve as preferred breeding sites for *P. orientalis*.

## Background

Visceral leishmaniasis (VL) or kala-azar is vector borne disease that is almost fatal if left untreated [1, 2]. More than 90% of VL cases in the world occur in Bangladesh, India, Nepal, Sudan, Ethiopia and Brazil [2]. *Phlebotomus martini* and *Phlebotomus orientalis* are the two principal vectors of kala-azar in East Africa [3, 4]. *Phlebotomus martini* is associated with *Macrotermes* termite mounds in southern Ethiopia [5] while *P. orientalis* is associated with *Acacia seyal - Balanites aegyptiaca* forest and deeply cracking soil in eastern Sudan and northwestern Ethiopia [6–18]. *P.orientalis* is believed to depend on the black cracking soil as day resting and breeding site in dry season [7, 8]. Elnaiem *et al*. [14] considered termite mound as principal day resting site for *P. orientalis* compared to tree holes, crevices, soil cracks and chicken coops in eastern Sudan. The breeding site of *P. orientalis* has not been identified beyond doubt as attempts to find larvae were not successful [7]. Further, the sugar feeding habits and diapauses of this species have not been described [7, 8]. The reservoir hosts of zoonotic VL (ZVL) in Eastern Africa Region are not known. Due to lack of knowledge on zoonotic transmission cycles and the ecology of the vector, little has been achieved in control of VL in the region. *A. seyal* – *B. aegyptiaca* forest was reported to have the highest *P. orientalis* population in Sudan [7, 12, 13, 15–17]. Elnaiem *et al*., [16] described *P. orientalis* as forest species with special preference to dense *A. seyal* than *B. aegyptiaca*, *Combretum kordofanum*, *Hyphaena* or *Zizipus* trees. The smooth surface of *A. seyal*, however, lacks cracks, fissures or cavities to serve as resting site during wet rainy season as oppose to *Balanites* sp. or other trees in the forest [7].

Metema–Humera lowlands in northwest Ethiopia are endemic for kala-azar and accounted 60% of all VL cases in Ethiopia [1]. Matema–Humera lowlands are, geographically, an extension of eastern Sudan [8, 15, 17] and have similar rainfall pattern and vegetation [7, 8]. The most kala-azar affected part of Matema–Humera lowlands is the Kafta-Humera district with the annual incidence that range from 1000 to 2000 cases, with higher prevalence (>80%) in labour migrants from Amhara and Tigray highland areas compared to the permanent residents in the area [19]. World Health organization report on leishmaniasis in tropical Africa [20] indicated that 45.6% of Humera population involved in farm activities were positive for leishmanin skin test compared to 8.3% in non-farmers (urban and farm-owning population) with annual sero-conversion rate of 7% and less than 1% respectively. Kala azar infections in labour migrants from non-endemic highland areas of Tigray and Amhara regions were addressed to the travel history to Kafta-Humera [21, 22]. Almost all (156/157 or 99.4%) kala azar cases in the labour migrant visiting Kafta Humera during June – October rainy season were aged from 15 to 49 compared to 68.9% (104/151) in permanent residents for the same age group (22) indicating this age group, that involved in agricultural activities in the extra-domestic habitats, as high risk group. Kala azar is the most important public health problem and cause high mortality and morbidity rate, especially among the young adult working forces, and has serious impact on the socio-economy of Kafta-Humera district. While treatment of kala-azar patients saves lives, it does not stop the disease from becoming a public health threat. Prevention of kala-azar transmission by vector control requires in depth understanding of the biology and ecology of sand fly vectors. Study on population dynamics and habitat preferences of *P. orientalis* are among the first steps in vector management to control kala-azar. Thus, the aim of this study was to describe the seasonal dynamics of *P. orientalis* and habitat preferences in extra-domestic habitats of the Kafta Humera lowlands.

## Methods

### Study area

**Figure 1:**
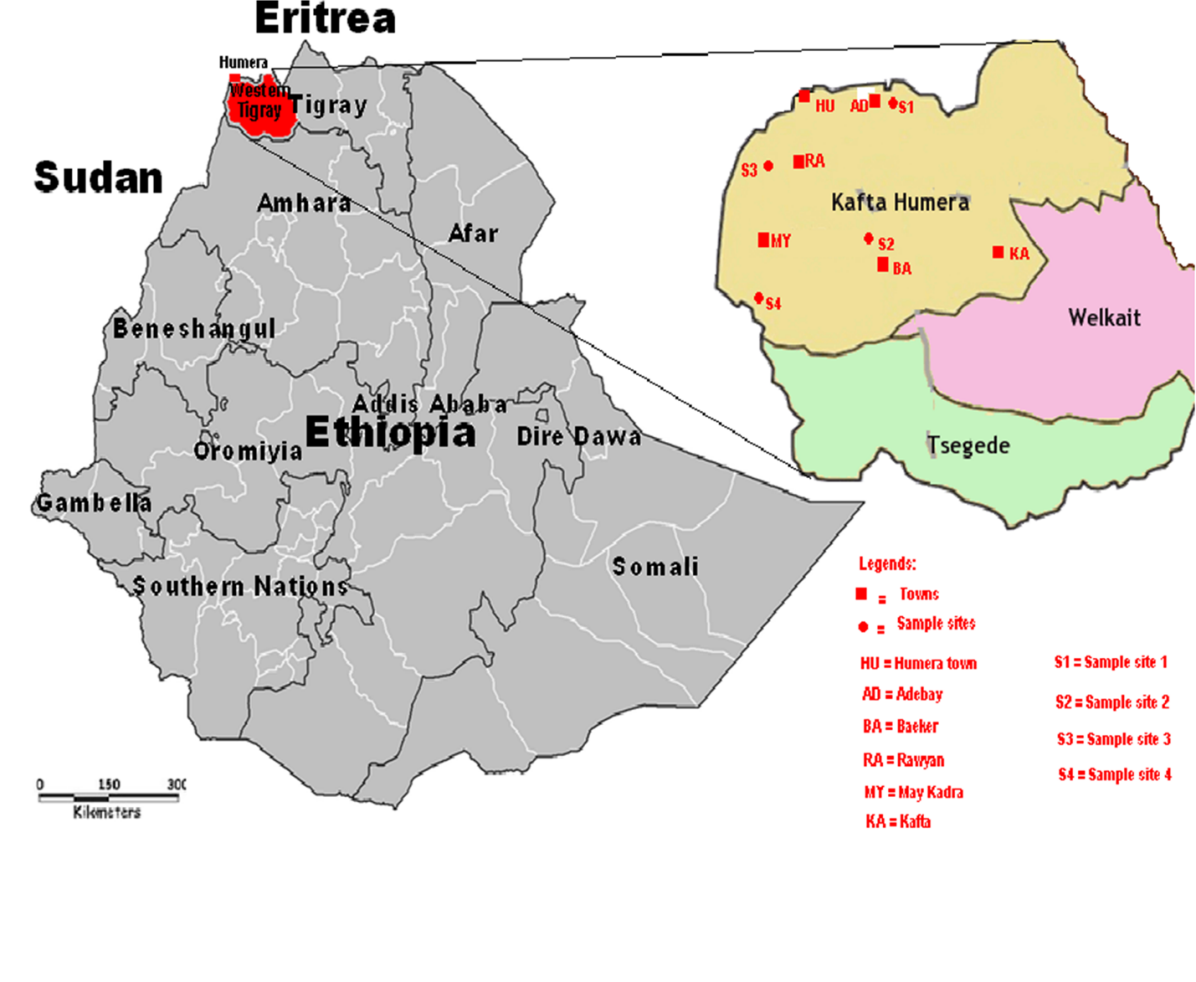
**Map of Western Tigray region indicating sand fly sampling sites in Kaft Humera district.**

Kafta Humera district (wereda) is found in Western Tigray Zone which includes Welkait and Tsegede districts (Figure 1). Humera town is the administrative center of the district. It has latitude 14°17′N and longitude 036°39′E at an altitude of 637 m above sea level. Repatriation of 14, 255 refugees from Saffawa and Umrakoba camps in eastern Sudan in 1993 and 1994 around Humera, resulted in an emergence of Rawyan (14°17′ 19″N, 036°37′ 18″E, 600 m a.s.l), May Kadra (14°08′ N, 036°34′ E, 612 m a.s.l), and Adebay (14°17′ 22 ″N, 036°38′E, 625 m a.s.l) towns. These settlements were after the end of the war in 1980’s and the Ethiopian People Democratic Front (EPDF) came to power. Before the settlements, about 720 people were living in May Kadra. The other town around Humera is Baeker which was a small village before Ethio-Eritrea war which displaced people from border areas around Humera and forced them to settle in Beaker. Epidemic of kala azar was erupted in 1995 [1], particularly in May Kadra (including Berket settlement center), which might be related to the movement of immunologically naïve people into kala azar endemic areas. Adebay is located 25 km away from Humera to eastern direction. May Kadra is a town located 25 km South of Humera. Rawyan is found between May Kadra and Humera. Baeker is located east of May Kadra at 40 km or southeast of Humera, at 53 km (Figure 1). Further east, towards Welkait district, at around 45 km from Baeker, is the Kafta town. Except the areas around Kafta town (above 1000 m a.s.l), all the lowlands (around 600 m a.s.l) in Kafta Humera district are endemic for kala azar. The district has a total population of 92,167(47,909 men and 44,258 women) and covers an area of 4,542.33 square km [23]. In Kafta Humera lowlands, the towns are surrounded by uniform agricultural fields that occasionally interrupted by thickets of *A. seyal* in depressions. All the dense mixed forests were converted into agricultural fields except in the part of Kafta Shiraro Park and some rocky outcrops.

### Study sites and habitats

**Figure 2:**
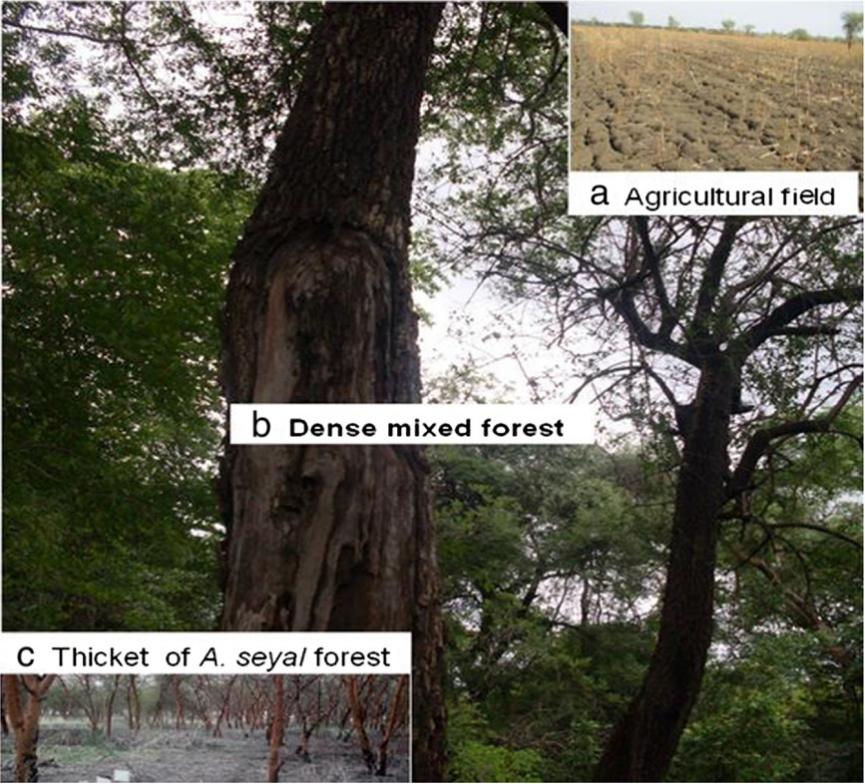
**Agricultural field (a), dense mixed forest (b) and thickets of** ***Acacia seyal*** **forest (c) habitats from where sand flies were sampled.**

A preliminary survey was conducted during field trip in May 15 – 28, 2010 to identify the habitats in the study area by the research team from Addis Ababa and Hebrew Universities. Sand fly sampling sites were selected from extra-domestic habitats areas around the Adebay (Site-1), Baeker (Site 2), and May Kadra (Site 4) towns where labour migrants perform agricultural activities during wet rainy season (Figure 2). The sampling sites were at least 10 km away from these towns. Site 4 was located 23 km south of the May Kadra in an area called Gelanzeraf closer to the Sudan boarder (13°59′N and 036°31′E). Each site had agricultural field, thicket(s) of *A. seyal* and dense mixed forest habitats (Figure 2) for sand fly sampling except Adebay and Rawyan where thicket of *A. seyal* and dense mixed forest were missing respectively. The shortest possible distance between two sampling sites (site 1 – site 3) was 35 km compared to 60 km longest distance (site 1- site 4). The agricultural fields that were used as sample sites were those which have been used for cultivation of sesame. All the thickets of *A. seyal* sample sites were found in depression where water floods during rainy season, next to agricultural fields. The thickets of *A. seyal* in around Rawyian town were dense (>20 trees/100 m^2^) compared to the sparse (10–20 trees/100 m^2^) in around the Baeker town or both thin (5–10 trees/100 m^2^) and sparse in Gelanzeraf area. The two ends of dense mixed forest (>20 trees/100 m^2^) extending between Adebay and Beaker towns (about 45 km) were used as extra-domestic sampling sites around Adebay (site 1) and Baeker (site 2). This forest is part of the Kafta Shiraro National Park - the largest park in Ethiopia. Another dense mixed forest with rocky and black soil was also used as sample site in Gelanzeraf area. The Common trees and shrubs in Kafta Humera areas are *A. seyal, A. mellifera, B. aegyptiaca*, *Terminalia spp*., *Boswellia papyrifera*, *Ficus sycomorus*, *Sclerocarya birrea*, *Zizypus spp*., *Dalbergia melanoxylon*, *Boscia angustifolia*, *Sterculia Africana, Adansonia digitata*, *Dichrostachys cineria* and *Syzgium guineese.* The field study and sand fly collection were conducted from May 17, 2011 to June 6, 2012.

### Climate

Information about mean average annual rainfall, mean maximum and minimum temperature of Humera and Baeker towns during January 2011 to December 2013 were obtained from Ethiopian National Meteorology agency to describe the climate of the study area.

### Sand flies collection and identification

At least 12 CDC - light traps/month (Model512, Hock and Co.,USA) were used to collect sand flies from agricultural fields, thickets of *A. seyal* and dense mixed forests at the four localities (sampling sites). Similarly, 20 – 388 sticky traps/month were used to collect sand flies from the three habitats. CDC light traps were set at 6 p.m, hanged at about 0.5 meter above ground level, and left overnight till 6 a.m. Sesame oiled sticky traps were randomly placed horizontally on the ground at about 5 m interval. Sand flies from sticky traps were collected in 95% alcohol before transferring to saline containing detergent for washing, sorting and counting *Sergentomyia* and *Phlebotomus* spp. Similarly, sand flies collected using CDC traps were sorted and counted. Sand flies were dissected in saline and mounted in Hoyer’s medium, after the head is separated and turned upside down before placing cover slip. The last segment of the abdomen was also removed for visualizing the spermathecae in female sand flies. Species identification was carried out using the appropriate keys [7, 24, 25].

### Study on habitat preference and population dynamics (Bionomics)

The sand fly species, sex, habitat, numbers, date and type of trap used were documented. Mean Monthly Density (MMD) of trapped sand flies was determined by total counts divided by number of traps used. Comparisons of *P. orientalis* MMD in different habitats were used for study of habitat preference of this vector. Similarly, MMD of *P. orientalis* at different months were used to determine the population dynamics. Resting sites of *P. orientalis* in the dense mixed forest during rainy season were determined by comparing the result of *P. orientalis* cached using the sticky traps placed on the ground and the emergence traps deployed on the tree trunks. Hand used torch light battery was also used for making observations of sand flies resting sites.

### Statistical analysis

The density of sand flies calculated as average numbers of male and female sand flies per trap per day and the results were entered into Statistical Package of Social Sciences (SPSS) version 16 for analysis of the data using descriptive statistics (Mean ± SD), analysis of variance (ANOVA) and Post hoc Tukey Honestly Significant Difference (HSD) tests so that seasonal dynamics and habitat preferences of *P. orientalis* could be studied. P-values less than 0.05 were considered as statistically significant difference.

## Results

### Climate: temperature and rainfall

**Figure 3:**
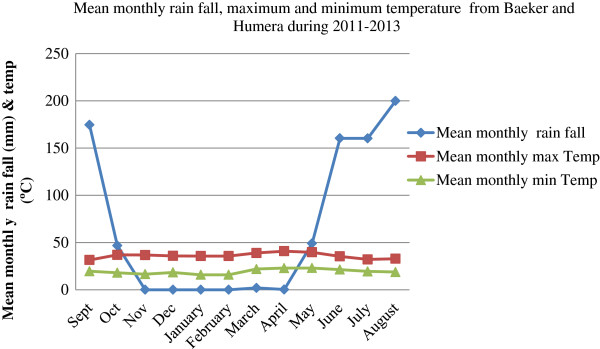
**Mean maximum (°C), Mean Minimum temperature (°C), and rain fall (mm) of Humera and Baeker towns from January 2011 to December 2013.**

The annual mean maximum temperature varied from 29.10 to 41.2°C while the monthly mean minimum from 13.50 to 25.40°C in Baeker and Humera towns (Figure 3). November to May was dry season and characterized by high mean maximum temperature (35.7 – 40.83°C), lack of heavy rain and cracking of black soil. Wet rainy season was found during June – October. The heaviest rain occurred in August (199.97 mm). The binging of rain was in May (49.08 mm), although this month remained very hot (39.66°C). The Average annual rainfall received by the area from January 2011 to December 2013 was 791 mm. March – May considered as hottest season (38.9 - 40.83°C) compared to October – February (35.7 – 36.88°C) and June - September rain season (31.5 - 35.33°C). Strong wind which started in the begging of rain (May) continued until it settled down in July. Strong winds affected the collection of sand flies in May and June. After the heaviest rains in August, depressions were flooded with water and rivers reached their peak height.

#### *Phlebotomus orientalis*density

A total of 376,441 sand flies were collected using CDC (n = 955) and sticky traps (n = 5551) from agricultural fields, thickets of *A.* seyal and dense mixed forests during the study period. Of the total sand flies collected using CDC and sticky traps (STs) from the three types of habitats, 62, 733 (5,546 female and 57,187 male) or 16.7% were *P. orientalis*. There was statistically significant difference between mean values of *P. orientalis/*traps for different sex (p = 0.001). The Mean ± SD of *P. orientalis/*CDC in all habitats was 8.03 ± 20.77 (range: 0–144.33) for male and 3.62 ± 7.01 (range: 0–49) for female. Similarly, the overall Mean ± SD of *P. orientalis/*sticky was 9.57 ± 27.07 (range: 0–295) for male and 0.32 ± 0.92(range: 0–11) for female. Almost all (99%) *Phlebotomus* spp found in the extra-domestic study sites were *P. orientalis* (Table 1).

**Table 1:** **Total sand flies collected from the agricultural fields, thickets of** ***A. seyal*** **and dense mixed forest using CDC and sticky traps from May 17, 2011 to June 6, 2012 G.C**

	CDC (No. traps (n) = 955)			Sticky (n = 5551)		
Species	Female	Male	Total	Female	Male	Total
*Phlebotomus orientalis*	3,876	8,512	12,388	1,670	48,675	50,345
*P. papatasi*	88	189	277	16	224	240
*P. duboscqi*	7	24	31	0	0	0
*P. bergeroti*	0	21	21	0	0	0
*P. rodhaini*	30	6	36	0	27	27
*P. martini*	0	10	10	0	0	0
*P. alexandri*	0	11	11	0	0	0
*Sergentomyia s*pp	90,078	74,314	164,392	92,383	56,280	148,663
Total	94,079	83,087	177,166	94,069	105,206	199,275

### Population dynamics

**Figure 4:**
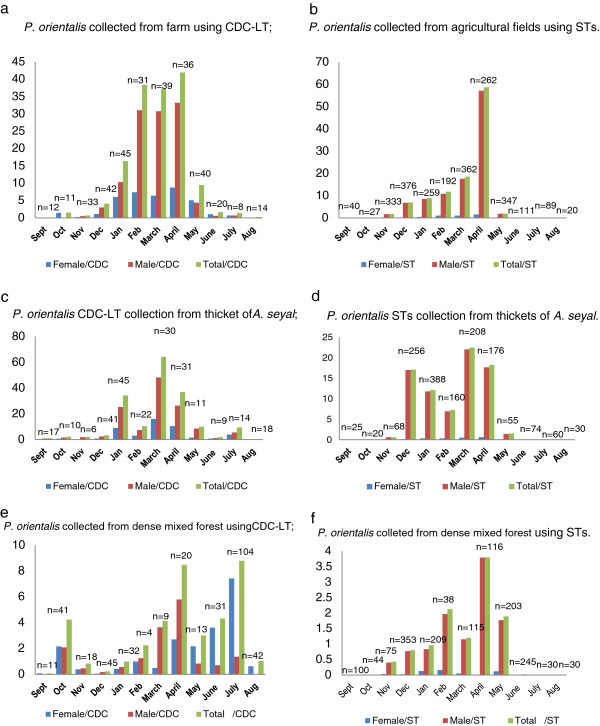
**Mean** ***P. orientalis/*** **trap in farm (a-b), thickets of** ***A. seyal*** **(c-d) and dense forest (e- f) habitats from May 2011 to June 2012.** n = no. traps used for each month.

For CDC traps, the highest MMD of *P. orientalis/*CDC was found in thickets of *A. seyal* in March (64.11 ± 75.87); and lowest was found in September (0 ± 0) in agricultural field. The highest *P. orientalis/*sticky was found in April (58.69 ± 85.20) in the agricultural field; and the lowest (0 ± 0) was found during July–October wet and rainy season in all habitats (Figure 4). When post hoc (HSD) tests were performed for mean *P. orientalis/*CDC in April, there was no significant mean difference with January (p = 0.384), February (p = 0.980) and March (p = 0.783) as opposed to the other months (p < 0.05). During rainy time, MMD of *P. orientalis/*CDC sharply decreased in June and July both in agricultural field and thickets of *A. seyal* compared to dense mixed forest which showed a highest MMD of *P. orientalis/*CDC (7.94 ± 10.50) in July with female: male ratio of 5.47:1. Dense mixed forest yielded 13.2% of the total *P. orientalis* collected from all habitats after using 392 CDC – traps or 39.4% of the total CDC traps used in this study. Of all *P. orientalis* (1577) collected from the dense mixed forest, 57.9% (772 female and 141 male) were trapped in July (8 *P. orientalis*/CDC) using 104 CDC traps (27.36% of the CDC traps used in dense mixed forest) (Figure 4).

### Habitat preference

The overall MMD of *P. orientalis* (female + male) trapped by CDC-LT was 15. 78 ± 28.93 (n = 320) in agricultural field, 19.57 ± 36.42(n = 255) in thickets of *A. seyal,* and 3.81 ± 6.45 (n = 380) in dense mixed forest. For STs, the overall MMD of *P. orientalis* was 14.76 ± 38.78 (n = 2378) in agricultural field, 11.45 ± 15.56 (n = 1500) in the thickets of *A. seyal* and 0.95 ± 2.16 (n = 1168) in dense mixed forest. ANOVA result has showed statistically significant mean difference (p = 0.000) for different habitats. However, similar habitats in different localities did not show statistically significant difference for the MMD of *P. orientalis* trapped by CDC-LT (p = 0.117) and ST (p = 0.134). During the August rains, habitat with more protection such as dense mixed forest, harbored more number of *P. orientalis/*CDC (1.04 ± 1.2) compared to agricultural fields (0.24 ± 0.42) and thickets of *A. seyal* (0.2 ± 0.44). Sticky traps placed on the ground in July and August did not collect sand flies when compared to emergence traps deployed on the tree trunks in the dense mixed forests. A total of 44 (32 female, 12 male) *P. orientalis* and 8115 *Sergentomyia sp.* (3736 female, 4379 male) were collected using 77 emergence traps.

Baeker sparse thickets of *A. seyal* (counts of 29.37 ± 49.41) and thin and sparse thickets in Gelanzeraf (with counts of 28.00 ± 41.33) were ranked first and second compared to the different habitats in different sites based on mean number of *P. orientalis*/CDC during the study period. The other habitats with higher mean *P. orientalis*/CDC values were agricultural fields in Baeker (counts of 22.47 ± 33.24) and Adebay (counts of 21.11 ± 34.07). ANOVA for mean number *P. orientalis*/CDC between sparse thickets of *A. seyal* in Beaker and sparse and thin thickets of *A. seyal* in Gelanzeraf showed no statistically significant difference (p > 0.05). Similarly, ANOVA for mean number *P. orientalis*/CDC in Adabay and Beaker agricultural fields showed no difference (p > 0.05). The mean number *P. orientalis*/CDC in the agricultural fields of Gelanzeraf (5.00 ± 9.57, n = 81) was very low compared to the thickets in the same area (28 ± 41.33, n = 149). The mean number *P. orientalis*/CDC of dense thickets of *A. seyal* (2.74 ± 3.64, n = 78) of Rawyan was as low as the agricultural field (1.57 ± 3.09, n = 49) in the same area. Generally, dense mixed forests in all sites also have low (3.81 ± 6.45, n = 357) mean *P. orientalis*/CDC during the study period and showed no statistically significant mean differences (p = 0.22) (Table 2). When mean *P. orientalis*/CDC for all thickets (19.37 ± 36.42, n = 248) and dense mixed forest (3.81 ± 6.45) were compared, there was statistically significant difference between these values (p = 000). But, no difference (p = 0.55) was observed between dense *A. seyal* in Rawyan (2. 86 ± 3.69, n = 83) and the dense mixed forests. Post hoc Tukey HSD test has showed sparse and thin Gelanzeraf thickets of *A. seyal* had almost the same mean number *P. orientalis*/CDC with sparse thickets of Beaker Site 2 (p = 0.994) but different mean counts when compared to dense Rawyan thickets of *A. seyal* (p = 0.036).

**Table 2:** ***P. orientalis*** **abundance in different sampling sites in relation to specific habitats, i.e., agricultural fields, thickets of** ***A. seyal*** **and mixed forest using CDC traps**

Locality	Agricultural field			Thickets of *A. seyal*			Dense mixed forest		
	*P. orientalis*/CDC			*P. orientalis*/CDC			*P. orientalis*/CDC		
	CDC	Mean	Std.D	CDC	Mean	Std.D	CDC	Mean	Std.D
Adebay extra domestic site 1	110	25.99	36.98	-	-	-	92	4.75	6.03
Baeker extra domestic site 2	78	22.47	33.24	31	29.37	49.41	149	4.60	8.14
Gelanzeraf extra domestic site 4	85	5.00	9.57	140	28.00	41.33	116	1.41	2.09
Rawyan extra domestic site 3	47	1.57	3.09	77	2.74	3.64			
Total	320	15.78	28.93	255	19.37	36.42	380	3.81	6.45

## Discussion

Three decades ago, the vegetations of Metema – Humera lowlands were described as *A. seyal* – *B. aegyptiaca* forest and *Argeissus* - *Combretum* savannah woodlands [26] which have now been converted mainly into big mechanized agricultural fields, especially in Kafta Humera district. Absence of *A. seyal – B. aegyptiaca* forests and termite mounds in the study areas in this district gave advantage to analyze fewer habitats such as agricultural fields and thickets of *A.* seyal in addition to dense mixed forests in the periphery of Kafta - Shiraro National Park. These habitats are distinctly separated from each other as opposed to habitats studied previously that had no demarcation between agricultural fields and the different types of forests [7, 14, 16].

In the highlands of Belessa valley in Ethiopia, the population of *P. orientalis* was reported to show no significant variation from September (rainy season) to April (dry season) [27]. In contrast, in Sudan, the numbers of *P. orientalis* captured using STs were reported to be few in the early dry season (January and February) and increased significantly in March until it reached peak number in April. The population of *P. orientalis* declined in May and June when rain commenced [7, 8]. Similarity in the result between numbers of *P. orientalis*/trap in Sudan and this study could be due to similar ecology shared between eastern Sudan and northwestern Ethiopia along the border areas [8, 16]. During the study of sand flies in the Dinder National Park next to Ethiopian border, *P. orientalis* showed a slight peak from December 1994 to February 1995 and then dropped in March – May, and then peaked suddenly in June 1995 in thickets of *A. seyal* [14]. In this study, however, *P. orientalis* was reached peak mean numbers in March and April with sudden dropped in May (Figure 4). The effect of wind in affecting sand fly collection in May and June should not be under estimated. The trend of seasonal dynamics of *P. orientalis* in dense mixed forest did not fully match the patterns in agricultural field and thickets of *A. seyal* (Figure 4). Generally, MMD in dense mixed forest was low compared to other habitats, but it showed an increase during dry season as other habitats until July. In July, MMD reached its peak value unlike other habitats where *P. orientalis* population declined. These results might be enough for the conclusion of dense mixed forest not to be the breeding site for *P. orientalis*. The seasonal changes in *P. orientalis* population in dense mixed forest might be due to the inter-habitat shift of this vector. During rain and wind stress season (May-June), *P. orientalis* from neighboring agricultural fields and thickets of *A. seyal* might have shifted to dense mixed forests where cracks and burrows in tree trunks serve them protected. The cooler temperature inside the forest, which has prevented soil crack formation, might have played a significant role for dense forests not to act as breeding site.

Due to the fact that most previous studies [7, 8, 14] have considered *P. orientalis* as forest species and owing to the lack of comparisons among different types of forests (thickets of *A. seyal*, *A. seyal* – *B. aegyptiaca* and dense mixed forests), the exact habitats of *P. orientalis* were not identified. The overall mean collections of *P. orientalis* usin*g* sticky traps from the three different habitats such as 11.50/sticky traps (n = 2418) from agriculture fields, 12.17/sticky ( n = 1500) from thickets of *A. seyal* and 1.08/sticky (n = 1633) from dense mixed forest in Kafta-Humera lowlands could be compared with similar study in Sudan [7]. The overall result of mean *P. orientalis/*sticky from the different forests in the Paloich areas in South Sudan 0.195/sticky trap (n = 100) [7] was similar with the result obtained from dense mixed forest in Kafta Humera districts. These results were lower than the overall results obtained from agricultural fields and thickets of *A. seyal* and might also suggest dense forests not to be breeding sites for *P. orientalis*. Habitats with cracks during dry season due to exposure to the heat of the sun such as agricultural fields and less dense thickets of *A. seyal* might be a breeding sites of *P. orientalis.* Males of *P. orientalis* collected from thickets of *A. seyal* and agricultural fields were found with unrotated genitalia (data not shown) indicating these habitats as places where this vector emerged from larvae. A mere absence of *P. orientalis* in CDC light traps set in forest habitats of Paloich area (Sudan) could not have been exclusively due to the failure of *P. orientalis* not being attracted to light as already described [7]. The absence of cracking type black soil or other breeding sites of *P. orientalis* were decisive. Dense forests could be devoid of soil cracks as already reported [8] and unsuitable for *P. orientalis* breeding. Habitats of *P. orientalis* were reported to have persistently lower normalized difference vegetation index (NDVI) value during the dry season and experienced more extreme dry and wet seasons than *P. orientalis* negative sites [15]. Our field observations in northwest Ethiopia concur with this. Typically, agricultural fields and thickets of *A. seyal* (sparse or thin), where *P. orientalis* was caught in abundance appeared to experience full exposure of sun during dry season whereas being covered with vegetation during rainy season. Agricultural fields and sparse or thin thickets of *A. seyal*, including *A. seyal - B. aegyptiaca* woodlands, could be a target for future control of kala-azar in the northwest Ethiopia and Sudan.

## Conclusion

Less dense thickets of *A. seyal* and agricultural fields in Kafta Humera lowlands, which are characterized by deep black cracking soil in dry season, are breeding and resting habitats for *P. orientalis*, compared to dense mixed forests where *P. orientalis* shelters temporarily during rainy season. Kala-azar infections in Kafta Humera lowlands might have been related with the visits or permanent settlement on the agricultural fields where tickets of *A seyal* often found in narrow depressions around the agricultural fields.

### Recommendations

Further studies on man – *P. orientalis* contact and parasite isolations from the vectors, human and reservoir hosts in extra-domestic habitats will show the exact areas where labour migrants are exposed to infection of kala-azar.
